# 2451 Views of African American parent-child dyads on the immunization neighborhood to improve HPV vaccination rates

**DOI:** 10.1017/cts.2018.270

**Published:** 2018-11-21

**Authors:** Jennifer Erves, Pamela C. Hull, Consuelo H. Wilkins

**Affiliations:** Vanderbilt University Medical Center

## Abstract

OBJECTIVES/SPECIFIC AIMS: To better understand African American (AA) parents and their adolescents perceptions towards the immunization neighborhood to improve HPV vaccination rates. METHODS/STUDY POPULATION: We conducted qualitative interviews among a purposive sample of 30 AA parent-child dyads. We engaged the community (community advisory boards, community organizations) in the design and implementation of this study. Before each interview, we provided participants a brief survey to assess acceptability of various vaccination settings (i.e., pharmacies, health departments, and schools). An inductive, qualitative content analysis approach was used to analyze the data, and a constant comparison method was used to compare codes for theme development. Descriptives (i.e., frequencies) were used to analyze survey data with the SPSS version 23 software. RESULTS/ANTICIPATED RESULTS: Findings demonstrate that many parents were willing to get their adolescents vaccinated at the health department (n=19) followed by the pharmacy (n=17). However, majority of parents were less willing to get their adolescent vaccinated at school (n=21). Mixed results were found for children with many having positive attitudes towards alternative settings (health department=21; pharmacy=14; school=16). Parents viewed the health department as being stigmatized and unclean for adolescent immunizations in general, while children were unsure of the difference between the health department and the medical home for the vaccine. Both parents and adolescents viewed the pharmacy as “too open” but would use it if a nurse administered the shot and had a good tracking system. Both also expressed strong feelings against school vaccinations, especially HPV vaccine shots. However, would consider for convenience or if administration was done by a nurse. DISCUSSION/SIGNIFICANCE OF IMPACT: Findings from this study provide intervention targets to improve access to HPV vaccination in alternative settings. It further demonstrates the importance of community engagement for the success of translational research, in which we will use it to disseminate this study’s findings. Ultimately, this study could play a role in shifting the traditional model of the HPV vaccine being provided solely in the medical home to improve HPV vaccination rates.

